# Tomatidine Attenuates Airway Hyperresponsiveness and Inflammation by Suppressing Th2 Cytokines in a Mouse Model of Asthma

**DOI:** 10.1155/2017/5261803

**Published:** 2017-11-21

**Authors:** Chieh-Ying Kuo, Wen-Chung Huang, Chian-Jiun Liou, Li-Chen Chen, Jiann-Jong Shen, Ming-Ling Kuo

**Affiliations:** ^1^Department of Microbiology and Immunology, Graduate Institute of Biomedical Sciences, College of Medicine, Chang Gung University, No. 259, Wenhua 1st Rd., Guishan Dist., Taoyuan City 33303, Taiwan; ^2^Graduate Institute of Health Industry Technology, Research Center for Food and Cosmetic Safety, Research Center for Chinese Herbal Medicine, College of Human Ecology, Chang Gung University of Science and Technology, No. 261, Wenhua 1st Rd., Guishan Dist., Taoyuan City 33303, Taiwan; ^3^Division of Allergy, Asthma, and Rheumatology, Department of Pediatrics, Chang Gung Memorial Hospital, Linkou, Guishan Dist., Taoyuan City 33303, Taiwan; ^4^Department of Nursing, Chang Gung University of Science and Technology, No. 261, Wenhua 1st Rd., Guishan Dist., Taoyuan City 33303, Taiwan; ^5^School of Traditional Chinese Medicine, Chang Gung University, No. 259, Wenhua 1st Rd., Guishan Dist., Taoyuan City 33303, Taiwan; ^6^Center for Traditional Chinese Medicine, Chang Gung Memorial Hospital, Linkou, Guishan Dist., Taoyuan City 33303, Taiwan

## Abstract

Tomatidine is isolated from the fruits of tomato plants and found to have anti-inflammatory effects in macrophages. In the present study, we investigated whether tomatidine suppresses airway hyperresponsiveness (AHR) and eosinophil infiltration in asthmatic mice. BALB/c mice were sensitized with ovalbumin and treated with tomatidine by intraperitoneal injection. Airway resistance was measured by intubation analysis as an indication of airway responsiveness, and histological studies were performed to evaluate eosinophil infiltration in lung tissue. Tomatidine reduced AHR and decreased eosinophil infiltration in the lungs of asthmatic mice. Tomatidine suppressed Th2 cytokine production in bronchoalveolar lavage fluid. Tomatidine also blocked the expression of inflammatory and Th2 cytokine genes in lung tissue. *In vitro*, tomatidine inhibited proinflammatory cytokines and CCL11 production in inflammatory BEAS-2B bronchial epithelial cells. These results indicate that tomatidine contributes to the amelioration of AHR and eosinophil infiltration by blocking the inflammatory response and Th2 cell activity in asthmatic mice.

## 1. Introduction

Allergic asthma is a complex chronic lung disease characterized by asthma with pulmonary inflammation, wheezing, coughing, and reversible airflow obstruction causing shortness of breath and breathing difficulties [1]. During acute asthma attacks, the patient has to urgently take medication to relieve excessive airway contraction and reduce breathing difficulties and suffocation [2]. Inhaled corticosteroids and bronchodilators are the mainstay controller therapy for asthma attacks. Oral medication with leukotriene antagonist or theophylline also decreases and prevents the onset of asthma symptoms [3]. Although corticosteroids are effective and considered generally safe for the treatment of asthma, many people are still concerned that long-term oral or inhaled corticosteroids cause many side effects [4]. Thus, asthma patients seek complementary and alternative medicine for improved outcomes.

Previous studies found that airway hyperresponsiveness (AHR) is an important factor stimulating bronchoconstriction in allergic asthma [5]. Th2 cells recognize allergic antigens and produce IL-4, IL-5, and IL-13 to induce eosinophil infiltration in the lung, goblet cell hyperplasia in the trachea, and airway remodeling in the clinical manifestations of asthma [6]. In addition, IL-4 and IL-13 can stimulate mucus hyperproduction in tracheal epithelial cells, which would obstruct the airway in asthmatic patients [7]. Th2 cell-associated cytokines also induce the differentiation of B cells into plasma cells, which would secrete more IgE for mast cell activation and the release of more inflammatory and allergic mediators [8]. Thus, blocking Th2 cell activity is regarded as an important regulator of the development of asthma.

Tomatidine is a steroidal alkaloid isolated from the skins and leaves of eggplant, potatoes, and tomatoes [9]. Many plants in the Solanaceae family release tomatidine and *α*-tomatine against fungal infections [10]. A previous study found that tomatidine inhibits the replication of *Staphylococcus aureus* [11]. Tomatidine also inhibits the invasion activity of A549 human lung adenocarcinoma cells and induces HL-60 human myeloid leukemia cell apoptosis [12, 13]. Another study found that tomatidine suppresses iNOS and COX-2 expression by blocking the NF-*κ*B pathway in LPS-stimulated RAW 264.7 cells [14]. The purpose of the present study was to determine whether tomatidine can improve the allergic inflammatory response in the airways in asthma, including suppression of Th2-associated cytokine levels, goblet cell hyperplasia, eosinophil infiltration, and AHR.

## 2. Materials and Methods

### 2.1. Animals

Six to eight-week-old female BALB/c mice (~20 g body weight) were purchased from the National Laboratory Animal Center in Taiwan. All mouse experiments were approved and performed according to the ethical guidelines of the Laboratory Animal Care Committee of Chang Gung University and Chang Gung University of Science and Technology (IACUC Approval number 2011-013). All mice were housed and maintained under conventional air (20 ± 2°C, 60 ± 10% humidity) on a 12 h dark/light cycle at the Animal Center of Chang Gung University.

### 2.2. Sensitization and Challenge with OVA and Tomatidine Treatment

Ovalbumin (OVA; Sigma, St. Louis, MO, USA) was emulsified with aluminum hydroxide (Thermo, Rockford, IL, USA) in normal saline and delivered intraperitoneally on days 1–3 and 14 as described previously [15]. Mice were challenged with 2% OVA using an ultrasonic nebulizer (DeVilbiss Pulmo-Aide 5650D, USA) on days 14, 17, 20, 23, and 27. All mice were randomly divided into six groups: normal control mice that were sensitized and challenged with normal saline (normal group), OVA-sensitized mice that were sensitized and challenged with OVA (OVA group), OVA-sensitized mice treated with 5 mg/kg prednisolone (P group), and OVA-sensitized mice treated with 0.5 mg/kg (T0.5 group), 1 mg/kg (T1 group), or 5 mg/kg (T5 group) tomatidine (Sigma). Tomatidine was given via a single intraperitoneal injection one hour before the challenge and AHR assay. Mice were sacrificed on day 29.

### 2.3. Measurement of AHR

On day 28, mice inhaled 0 to 40 mg/ml methacholine for 3 min and put into the whole-body plethysmography chamber (Buxco Electronics, Troy, NY, USA) to record the data as an enhanced pause (Penh) as described previously [16]. To assay respiratory resistance, mice were anesthetized and intubated to measure respiratory input impedance in the presence of increasing doses of methacholine (0–30 mg/ml) using the low-frequency forced oscillation technique (Buxco Electronics). The result would represent airway resistance.

### 2.4. Histological Analysis of Lung Tissue

Lung tissue was embedded in paraffin and cut into 6 *μ*m sections. The slides were stained with periodic acid-Schiff (PAS) stain (Sigma) to measure goblet cell hyperplasia or hematoxylin and eosin (HE) to assay eosinophil infiltration as described previously [17].

### 2.5. Cell and Supernatant Collection in Bronchoalveolar Lavage Fluid

On day 29, bronchoalveolar lavage fluid (BALF) was collected from mice as described previously [18]. Briefly, the trachea was flushed with normal saline and cells stained with Liu stain solution (Polysciences Inc., Taipei, Taiwan) to measure the cell number and cell types in the BALF. Cytokines and chemokines were assayed in the supernatants by ELISA.

### 2.6. Serum Collection and Splenocyte Cultures

Mice were anesthetized and serum was collected to assay OVA-specific antibodies. Splenocytes were cultured with 100 *μ*g/ml OVA in RPMI 1640 medium for 5 days. Cytokine production was measured in the supernatants as described previously [18].

### 2.7. RNA Isolation and Real-Time PCR

Lung tissue was homogenized and total RNA isolated using Trizol reagent (Invitrogen, Paisley, Scotland) as described previously [19]. The cDNA was synthesized using the cDNA synthesis kit (Invitrogen) and gene expression measured using a spectrofluorometric thermal cycler and the SYBR Green Supermix (Bio-Rad Laboratories, Hercules, CA, USA).

### 2.8. Culture and Tomatidine Treatment of BEAS-2B Cells

BEAS-2B cells treated with tomatidine (3–100 *μ*M, dissolved in DMSO) for 1 h were cultured with 20 ng/ml TNF-*α* and 5 ng/ml IL-4 for 24 h. Cytokine and chemokine productions were measured in the supernatants using ELISA kits.

### 2.9. ELISA

ELISA kits and the supernatants were used to measure the levels of IL-4, IL-5, IL-6, IL-8, IL-13, IFN-*γ*, CCL11, and intercellular adhesion molecule 1 (ICAM-1) according to the manufacturer's instructions (R&D Systems, Minneapolis, MN, USA). OVA-IgE levels were detected in serum diluted 10-fold using the IgE-specific ELISA kit (BD Biosciences) by OD_450_.

### 2.10. Statistical Analysis

All data were assessed using one-way analysis of variance (ANOVA) and the Tukey‐Kramer post hoc test for comparisons among treatment groups. *P* < 0.05 was considered a significant difference.

## 3. Results

### 3.1. Tomatidine Attenuated AHR in Asthmatic Mice

**Figure 1:**
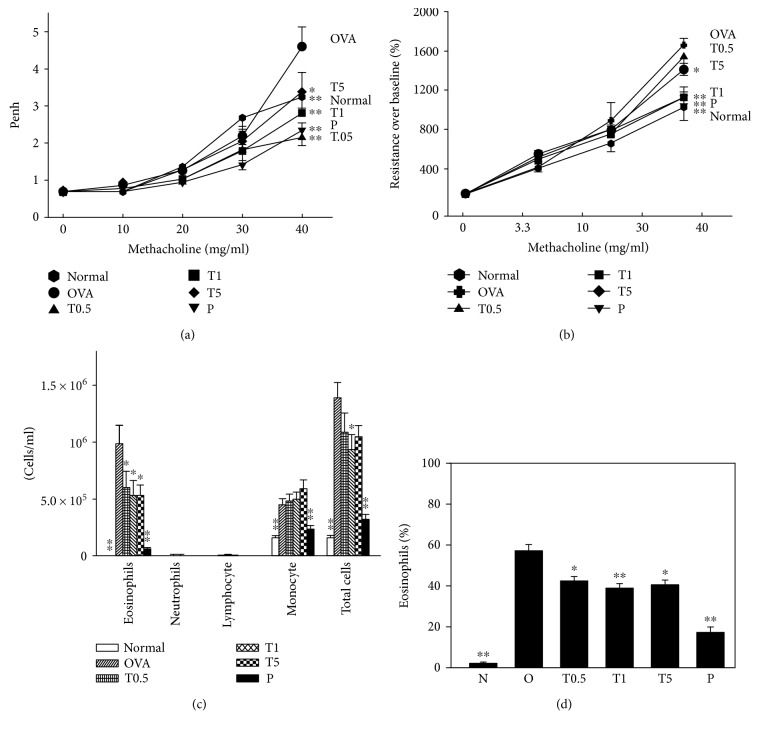
Tomatidine reduces airway hyperresponsiveness (AHR) in asthmatic mice. (a) Mice inhaled increasing doses of methacholine, and AHR was assessed and shown as Penh values. *n* = 12 mice/group in three independent experiments. (b) AHR was measured as a percentage from the baseline level of lung resistance. Effect of tomatidine on cell counts in bronchoalveolar lavage fluid (BALF) from asthmatic mice. (c) Inflammatory cells and total cells were counted in BALF. (d) Eosinophil percentages were measured in BALF. All data are presented as means ± SEM. ^∗^*P* < 0.05, ^∗∗^*P* < 0.01 compared to the OVA control group.

In order to determine whether tomatidine treatment affects AHR, airway mechanics were measured using a whole-body plethysmography chamber to record the signal as an enhanced pause (Penh). When mice inhaled 40 mg/ml methacholine, the groups treated with tomatidine had suppressed Penh values compared to the OVA group (Figure 1(a)). Airway resistance as determined by the forced intubation technique was significantly higher in the OVA group than the control group at 30 mg/ml inhaled methacholine. Treatment with tomatidine significantly decreased the airway resistance compared to the OVA group (Figure 1(b)). These results support tomatidine improving AHR during the development of an allergic reaction in asthmatic mice.

### 3.2. Tomatidine Prevents Inflammatory Cell Infiltration in BALF

During the development of asthma, an important biological marker is the antigen-induced increase in the number of inflammatory cells in the BALF. Tomatidine significantly attenuated the number of total cells and eosinophils compared to the OVA group (total cells: T0.5, 1.1 × 10^6^ ± 5.8 × 10^4^, *P* < 0.01; T1, 9.3 × 10^5^ ± 7.8 × 10^4^, *P* < 0.01; T5, 1.0 × 10^6^ ± 5.6 × 10^4^, *P* < 0.01; OVA, 1.4 × 10^6^ ± 1.6 × 10^5^; eosinophils: T0.5, 6.0 × 10^5^ ± 4.7 × 10^4^, *P* < 0.01; T1, 5.3 × 10^5^ ± 4.9 × 10^4^, *P* < 0.01; T5, 5.3 × 10^5^ ± 3.3 × 10^4^, *P* < 0.01; OVA, 9.8 × 10^5^ ± 6.1 × 10^4^) (Figure 1(c)). Tomatidine also significantly suppressed the percentage of eosinophils compared to the OVA group (Figure 1(d)).

### 3.3. Tomatidine Decreased Eosinophil Infiltration and Goblet Cell Hyperplasia in the Lungs of Asthmatic Mice

**Figure 2:**
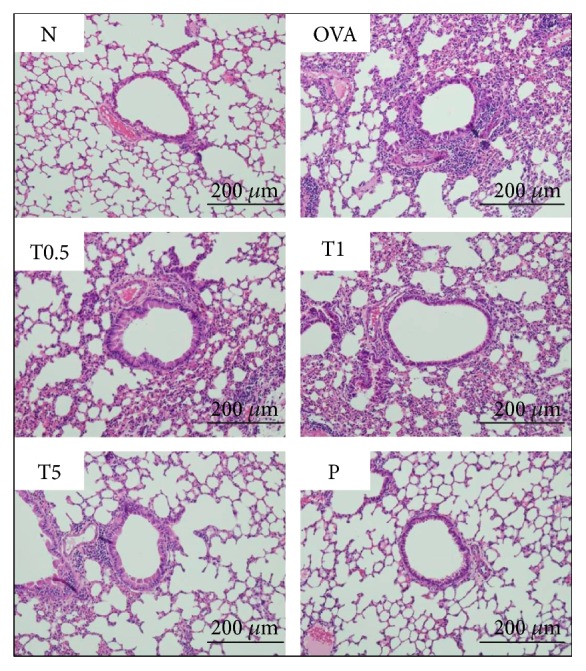
Effect of tomatidine on eosinophil infiltration (HE staining) in lung tissue (200x magnification).

**Figure 3:**
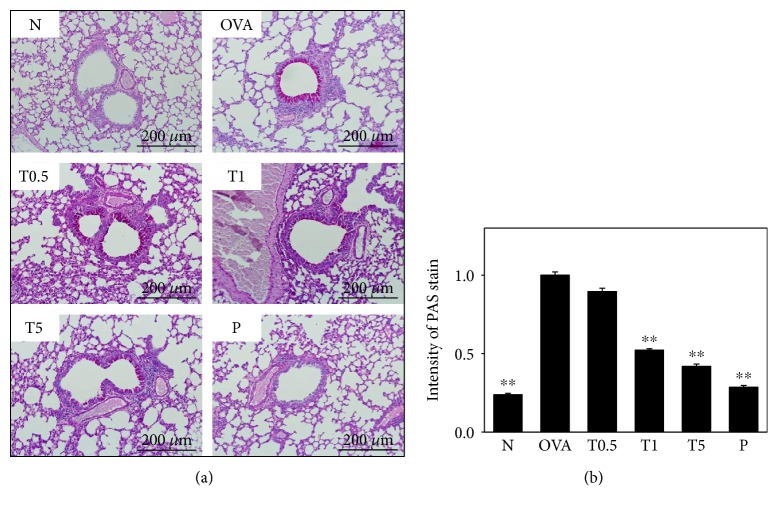
(a) PAS-stained lung sections show goblet cell hyperplasia in lung tissue (200x magnification). (b) Results expressed as the intensity of PAS stain. All data are presented as means ± SEM. ^∗∗^*P* < 0.01 compared to the OVA control group.

In OVA-sensitive mice, more eosinophil infiltration occurred between the blood vessels and bronchus compared to control mice. Asthmatic mice treated with tomatidine had significantly suppressed eosinophil infiltration in the lung tissue section (Figure 2). Furthermore, inflammatory goblet cell could aggravate mucus secretion to obstruct the airways. We also found that tomatidine significantly decreased goblet cell hyperplasia compared to OVA-sensitive mice (Figures 3(a) and 3(b)).

### 3.4. Effect of Tomatidine on Cytokine and Chemokine Levels in BALF

**Figure 4:**
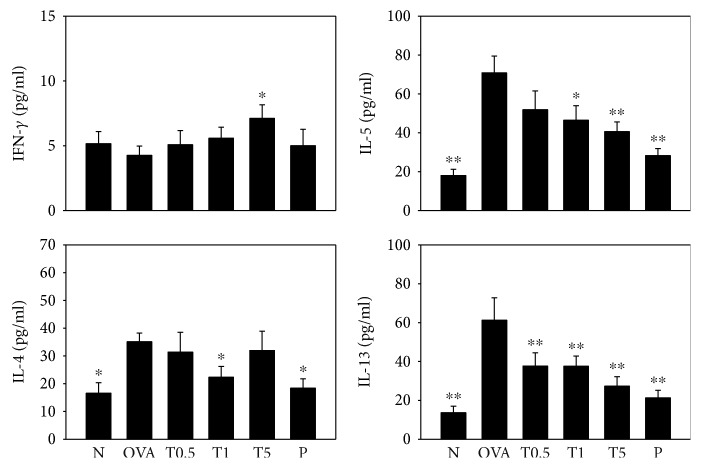
Effects of tomatidine on cytokine levels in bronchoalveolar lavage fluid. The concentrations of IFN-*γ*, IL-4, IL-5, and IL-13 were measured by ELISA. All data are presented as means ± SEM. ^∗^*P* < 0.05, ^∗∗^*P* < 0.01 compared to the OVA control group.

High-dose tomatidine (T5 groups) could increase IFN-*γ* levels and decrease IL-5 and IL-13 levels compared to the OVA group. However, only the T1 group could inhibit IL-4 production in BALF from asthmatic mice (Figure 4).

### 3.5. Effect of Tomatidine on Gene Expression in the Lung

**Figure 5:**
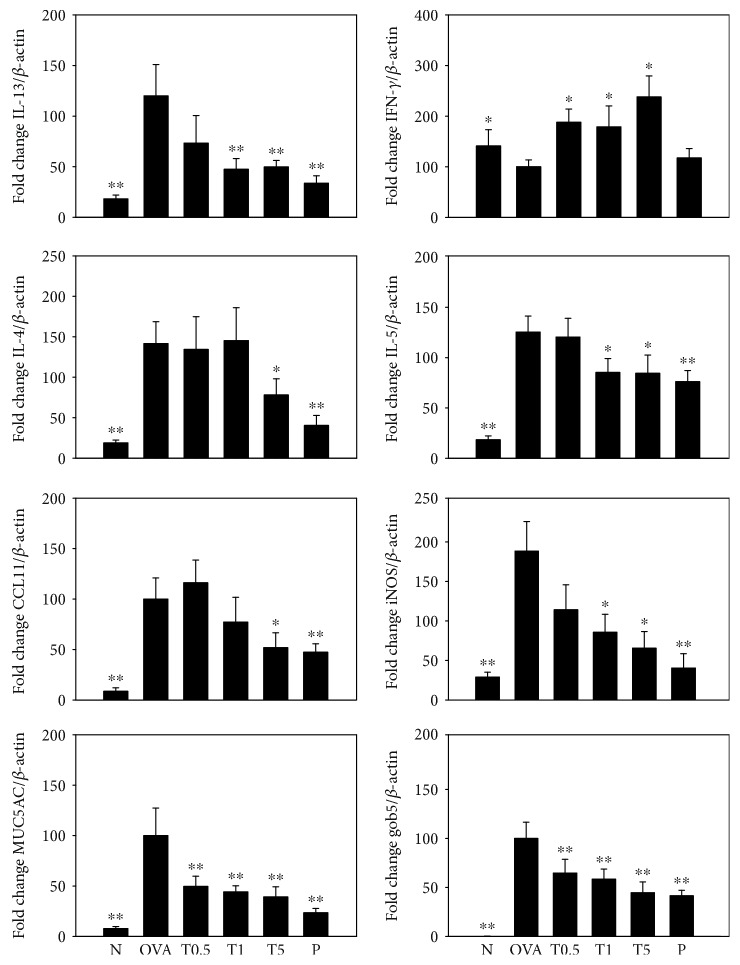
Effects of tomatidine on gene expression in the lung. IL-4, IL-5, IL-13, IFN-*γ*, CCL11, iNOS, MUC5AC, and gob5 expression was determined using real-time RT-PCR. Data are presented as means ± SEM. ^∗^*P* < 0.05, ^∗∗^*P* < 0.01 compared to OVA mice.

We used real-time PCR to measure gene expression in the lungs of asthmatic mice. Tomatidine significantly decreased IL-4, IL-5, IL-13, CCL11, iNOS, MUC5AC, and gob5 gene expression in OVA-sensitized mice and increased the expression of the IFN-*γ* gene compared to OVA-sensitized mice (Figure 5).

### 3.6. Effect of Tomatidine on Cytokine Levels in Splenocyte Culture and OVA-IgE Production in Serum

**Figure 6:**
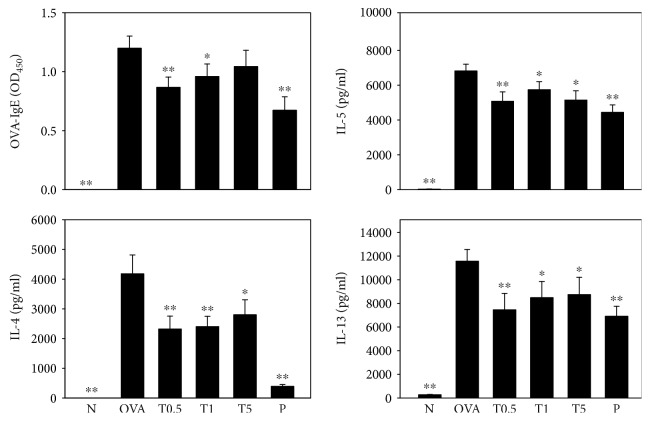
Tomatidine affected the levels of OVA-IgE in serum and IL-4, IL-5, and IL-13 in splenocyte culture supernatant. All data are presented as means ± SEM. ^∗^*P* < 0.05, ^∗∗^*P* < 0.01 compared to the OVA control group.

In supernatants from splenocyte culture, tomatidine significantly decreased IL-4, IL-5, and IL-13 production compared to the OVA group. In serum, treatment with 0.5 mg/kg and 1 mg/kg, but not 5 mg/kg, tomatidine significantly suppressed OVA-IgE levels (Figure 6).

### 3.7. Effect of Tomatidine on Inflammatory Mediators in BEAS-2B Cells

**Figure 7:**
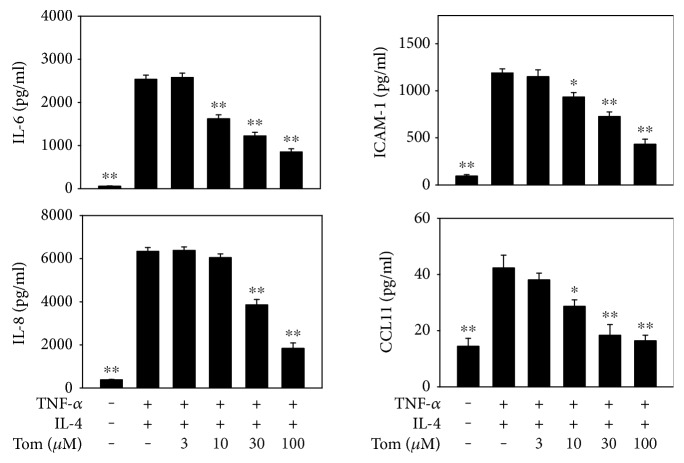
Tomatidine suppressed cytokine and chemokine levels in BEAS-2B cells. BEAS-2B cells treated with the indicated concentrations of tomatidine (Tom) for 1 h were cultured with 20 ng/ml TNF-*α* and 5 ng/ml IL-4 for 24 h. The supernatant was collected and the levels of IL-6, IL-8, ICAM-1, and CCL11 were measured by ELISA. All data are presented as means ± SEM. ^∗^*P* < 0.05, ^∗∗^*P* < 0.01 compared to the OVA control group.

BEAS-2B cells were activated with IL-4 and TNF-*α* to assess the anti-inflammatory effect of tomatidine. Tomatidine significantly decreased IL-6 and IL-8 levels compared to the TNF-*α*/IL-4 control group (IL-6: tomatidine 3 *μ*M, 2025.8 ± 238.1 pg/ml, *P* = 0.24; tomatidine 10 *μ*M, 1662.1 ± 234.7 pg/ml, *P* < 0.05; tomatidine 30 *μ*M, 1323.1 ± 123.1 pg/ml, *P* < 0.01; tomatidine 100 *μ*M, 1101.6 ± 175.4 pg/ml, *P* < 0.01; TNF-*α*/IL-4 control, 2122.8 ± 221.1 pg/ml; IL-8: tomatidine 3 *μ*M, 6184.5 ± 454.3 pg/ml, *P* = 0.31; tomatidine 10 *μ*M, 6096.3 ± 495.3 pg/ml, *P* = 0.22; tomatidine 30 *μ*M, 5274.3 ± 594.6 pg/ml, *P* < 0.05; tomatidine 100 *μ*M, 3472.3 ± 531.7 pg/ml, *P* < 0.01; TNF-*α*/IL-4 control, 6096.1 ± 425.3 pg/ml) (Figure 7). Tomatidine also significantly reduced ICAM-1 and CCL11 levels compared to the TNF-*α*/IL-4 control group.

## 4. Discussion

Asthma is a chronic inflammatory disease of the airways, and its incidence has increased in developed and developing countries [2]. An acute asthma attack would exacerbate smooth muscle contraction in the airway, causing severe shortness of breath [1]. Mucus hypersecretion in the airway obstructs airflow into the respiratory tract, causing breathing difficulties, suffocation, and even death [20]. Bronchodilators and inhaled steroids are common drugs for the alleviation of asthma attacks [21]. However, some patients still seek alternative therapies to improve asthma symptoms, including acupuncture, homeopathy, and Chinese herbal medicine [22, 23].

Previous studies reported that some secondary compounds in plants can attenuate AHR, eosinophil infiltration, and immunoregulatory effects in asthmatic mice [24, 25]. Anthocyanidins, flavonols (quercetin and kaempferol), flavones (apigenin and luteolin), and isoflavones (genistein) have been reported to ameliorate airway remodeling and the inflammatory response in mice [26, 27]. Phytosterol, *β*-sitosterol, and sitostanol have also been shown to suppress Th2 cytokine production and AHR in mice [28]. In the present study, tomatidine significantly inhibited eosinophil infiltration into the lung tissue and suppressed goblet cell hyperplasia in the trachea of asthmatic mice. Tomatidine suppressed AHR and decreased the levels of Th2-associated cytokines and chemokines in BALF. However, asthmatic mice treated with low-dose tomatidine (T1 group) had inhibited OVA-IgE production and decreased levels of Th2 cytokines (IL-4, IL-5, and IL-13) in splenocyte culture. Tomatidine significantly decreased the levels of chemokines (including IL-8 and CCL11), proinflammatory cytokines (IL-6), and cell adhesion molecules (ICAM-1) in human tracheal epithelial cells. Evidently, tomatidine improves the pathological manifestation and lung inflammatory response of asthma.

Th2 cells could release more cytokines to induce allergic and inflammatory cell infiltration into airways and lung tissue in asthma patients [29]. AHR is an important pathological and clinical characteristic in the underlying severity of asthma [27]. IL-13 knockout mice were found to suppress the aggravated AHR in allergic asthmatic mice [30]. Asthmatic mice that inhaled the anti-IL-13 antibody Fab′ fragment have been reported to have decreased AHR, airway inflammation, and remodeling [31]. In addition, asthmatic patients treated with anti-IL-13 antibody by intravenous infusions have improved pulmonary function [32]. We used a whole-body plethysmography chamber to measure the Penh value and found that tomatidine can suppress AHR in asthmatic mice. We also found that tomatidine decreases respiratory resistance, improving AHR in asthmatic mice. IL-13 levels were significantly decreased in BALF and splenocyte culture medium, and IL-13 gene expression was inhibited by tomatidine treatment in asthmatic mice. Thus, we thought that tomatidine could suppress Th2 cell secretion of IL-13, blocking AHR in mice.

IL-5 induces the differentiation and proliferation of eosinophils from hematopoietic stem cells in the bone marrow [7]. T cells and inflammatory tracheal epithelial cells released more eotaxins (CCL11 and CCL24) to induce mature eosinophil migration into the airways. Some studies have found that IL-5 receptor (IL-5Ra) knockout mice have induced allergic asthma without significant promotion of eosinophil infiltration [29]. Other studies have shown that asthmatic mice treated with anti-IL-5 have decreased AHR and eosinophil infiltration [33]. Tomatidine decreased IL-5 levels in BALF and splenocyte culture medium, and real-time analysis found that tomatidine decreased IL-5 gene expression in lung tissue from asthmatic mice. In addition, tracheal epithelial cells treated with tomatidine had suppressed CCL11 production and reduced ICAM-1 levels, decreasing eosinophil adhesion. Thus, tomatidine could effectively reduce eosinophil infiltration into the lungs of asthmatic mice. In infective or inflammatory tissues, activated eosinophils release eosinophil cationic protein and eosinophil peroxidase, causing injury and inflammatory responses in the lung [34]. Inflammatory lung macrophages express iNOS, which catalyzes arginine for nitric oxide production [35]. Nitric oxide stimulates lung macrophages to release more inflammatory mediators and cytokines, causing more severe lung inflammation in asthmatic mice [36, 37]. Tomatidine decreased iNOS gene expression, inhibiting lung inflammation. In addition, inflammatory BEAS-2B cells treated with tomatidine had reduced chemokine and proinflammatory cytokine levels, inhibiting local airway inflammation in asthmatic mice. Thus, tomatidine had a good effect, alleviating airway inflammation by inhibiting inflammatory cytokines, chemokines, and eosinophil infiltration of the lung.

In asthmatic patients, goblet cells in the trachea are more hyperplastic and excessively secrete mucus to obstruct airways [38]. Mucin is a family of glycoproteins produced by epithelial and endothelial cells [39]. Goblet cells in the airway also release mucin against allergens or microbes that enter the respiratory system. Gob5, a member of the calcium-activated chloride channel family, could increase goblet cell hyperplasia in asthmatic mice. Gob5 knockout mice have decreased mucus production, and gob5 blocks AHR in OVA-sensitive mice [40]. IL-13 and IL-4 could stimulate tracheal goblet cell hyperplasia and increase mucin secretion [32, 39]. We found that tomatidine decreases IL-4 and IL-13 levels in BALF and splenocyte culture medium and inhibits IL-4 and IL-13 gene expression in the lung tissue of asthmatic mice. Tomatidine also significantly decreases goblet cell hyperplasia in the airways and suppresses gob5 and mucin5AC gene expression in asthmatic mice. We concluded that tomatidine decreases goblet cell hyperplasia and reduces mucus production that obstructs airways and causes breathing difficulties by blocking IL-13 and IL-4 production for gob5 and mucin5AC expression in OVA-sensitized mice. IL-4 also stimulates B cell IgE productions [7]. Allergy-IgE antibodies bind to the Fc*ε*R1 receptors of mast cells, inducing histamine and leukotriene release, causing a violent hypersensitivity and inflammatory response in airways [7, 41]. We concluded that tomatidine suppresses IgE production in the serum by blocking IL-4 expression in asthmatic mice.

In this study, we confirmed that tomatidine can improve asthma symptoms in mice. Tomatidine significantly suppresses mucin production, airway inflammation, AHR, and eosinophil infiltration by blocking Th2 cell activity in asthmatic mice.
